# Auditory conditioned fear elicits anxiety-like behavior and differential neuronal remodeling in the prelimbic and infralimbic cortex of rats

**DOI:** 10.1007/s00429-026-03124-6

**Published:** 2026-06-22

**Authors:** Iris Dalia Vázquez-Vargas, Gonzalo Flores, Julio César Morales-Medina, Linda Garcés-Ramírez, Fidel De la Cruz-López

**Affiliations:** 1 Departamento de Fisiología, Escuela Nacional de Ciencias Biológicas, Instituto Politécnico Nacional, Unidad Profesional “Adolfo López Mateos”, Av. Wilfrido Massieu s/n, Ciudad de México, 52333 México; 2 Instituto de Fisiología, Benemérita Universidad Autónoma de Puebla (BUAP), Prol. de la 14 Sur 6301, Ciudad Universitaria, 72592 Heroica Puebla de Zaragoza, México; 3 Centro de Investigación y de Estudios Avanzados del Instituto Politécnico Nacional, Unidad Tlaxcala, Ixtacuixtla, 90120 Tlaxcala, México

**Keywords:** Auditory conditioned fear, Dorsal immobility test, Infralimbic cortex, Open field test, Prelimbic cortex

## Abstract

Fear and anxiety are adaptive responses that promote survival. Defensive immobility, including tonic and dorsal forms, is a multisensory behavior. However, the relationship between learned fear and immobility, as well as underlying cortical adaptations, remains unclear. Here, we induced an auditory conditioned fear in adult rats and assessed its effects on locomotion, light/dark preference, and two immobility paradigms, alongside neuroarchitectural changes in the medial prefrontal cortex including the prelimbic (PrL) and infralimbic (IL) cortices. Conditioned rats spent significantly less time in the light compartment and more time in the dark compartment of the light/dark box. In the open-field test, they exhibited reduced distance traveled and increased freezing behavior. Dorsal immobility duration was elevated in conditioned rats, whereas neck-clamp immobility remained unaffected. Morphological analyses revealed region-specific alterations in prefrontal cortical structure: conditioned rats displayed hypertrophy in the PrL cortex, while the IL cortex showed hypotrophy and reduced spine density. These opposing, region-dependent changes suggest differential cortical adaptations associated with learned fear. Together, these results indicate that conditioned rats display behavioral alterations associated with anxiety-related behavior accompanied by selective neuroarchitectural remodeling within prefrontal circuits implicated in fear regulation. Our findings provide novel insights into the interplay between conditioned fear, defensive immobility, and cortical plasticity, highlighting distinct roles of PrL and IL subregions in modulating adaptive and maladaptive responses to threat.

## Introduction

Fear and anxiety are adaptive behavioral and physiological responses that promote survival in threatening situations. While anxiety functions as an anticipatory state preparing the organism for fight-or-flight, phobias are characterized by excessive and irrational fear responses often resulting from Pavlovian conditioning, where a neutral cue becomes associated with an aversive unconditioned stimulus (Giustino and Maren 2015; Ohi et al. 2025). The immobility reflex, or “freezing,” involves a temporary cessation of movement and responsiveness when escape is impossible (Korczynski and Korda 1988). This reflexive state may offer adaptive advantages by reducing detectability and minimizing injury during predatory encounters. Fear regulation relies on a distributed neural network encompassing the medial prefrontal cortex (mPFC), amygdala, and hippocampus (Sierra-Mercado et al. 2011; McEwen and Morrison 2013; Moustafa et al. 2013). Within the mPFC, the infralimbic (IL) and prelimbic (PrL) regions—homologous to Brodmann areas 25 and 32 in primates—exert opposing roles in conditioned fear (McEwen and Morrison 2013). A functional dichotomy has been proposed between these two cortices in the regulation of different aspects of fear (Vidal-Gonzalez et al. 2006; Sierra-Mercado et al. 2011; Giustino and Maren 2015). Alterations in these circuits may contribute to maladaptive fear states such as phobias or anxiety disorders.

Among behavioral expressions of defensive reactions, the immobility response (IR) represents a distinctive phenomenon characterized by profound stillness and reduced responsiveness. IR is typically induced by positioning the animal in an unnatural posture maintained by manual restraint and involves multimodal sensory input, including tactile, proprioceptive, vestibular, and occasionally visual stimuli (Klemm 1971; Lalonde and Strazielle 2022). Importantly, IR does not occur spontaneously but rather arises as a reaction to environmental cues indicative of restraint. Pharmacological evidence indicates that dopaminergic antagonists and opioids potentiate IR (Klemm 1989; Lalonde and Strazielle 2022), suggesting that dopaminergic and opioid systems contribute to the modulation of this behavior.

While all IR paradigms share core features, variability in induction methods across laboratories has complicated direct comparisons and interpretations (Klemm 1971; Crawford 1977; Webster et al. 1981; Lalonde and Strazielle 2022). Nevertheless, distinctions among these paradigms have provided insight into the sensory triggers and neurobehavioral mechanisms underlying immobility. One of the most extensively studied forms is tonic immobility (TI), which is typically induced by placing an animal in a supine position. This form of immobility is readily observed in species such as guinea pigs, rabbits, and frogs but is difficult to elicit in adult rats (Liberson et al. 1961; Klemm 1971). In contrast, neonatal rats display pronounced TI, and its duration can be significantly increased by haloperidol, a dopamine receptor antagonist, at postnatal day 10 but not at later developmental stages (Meyer et al. 1984). This developmental pattern suggests a transient sensitivity of dopaminergic systems in the modulation of immobility early in life.

Another well-characterized variant is the dorsal immobility response (DIR), elicited by grasping the skin on the dorsal surface of the neck and lifting the animal into the air (Webster et al. 1981; Korczynski and Korda 1988; Pellis et al. 1990). DIR occurs in several species, including rabbits, chickens, guinea pigs, and rats. In 20-day-old rats, haloperidol potentiates DIR but not TI (Meyer et al. 1984), whereas in adults, haloperidol enhances DIR duration but reduces it in neonates (Wilson et al. 1984). These findings indicate developmental and neurochemical differences in the regulation of immobility responses, likely reflecting maturation of dopaminergic pathways and sensory integration mechanisms.

A third form, clamp-induced immobility, is produced by applying pressure to the nape of the neck while positioning the animal in a supine, ventroflexed posture with limbs drawn inward (de-la-Cruz et al. 1987; de la Cruz and Junquera 1989; de la Cruz et al. 1990). The effective stimulus in this paradigm is tactile pressure on the nuchal skin, which can be applied manually, via a clamp, or naturally by the teeth of an adult rat acting on neonates (Brewster, 1980). Neonatal rats are particularly sensitive to this form of restraint, and a similar effect can be achieved by wrapping a bandage around the head and neck—a technique referred to as “bandaging” (de-la-Cruz et al. 1987). Adult rats, however, remain responsive to DIR and clamp-induced IR but not to bandaging, suggesting that developmental changes in sensory processing modulate susceptibility to different forms of restraint (de-la-Cruz et al. 1987). Across paradigms, immobility behaviors share several similarities with opioid-induced states, such as inhibition of righting reflexes, suppression of clinging or escape behaviors, and alternation between quiescent and active escape phases (Costall and Naylor 1974; Gallup 1974; De Ryck et al. 1980; Pellis et al. 1986). These parallels suggest that common neural substrates, possibly involving endogenous opioid modulation and dopaminergic inhibition, contribute to the generation and maintenance of immobility states.

Collectively, existing evidence supports the notion that IR represents a complex, multisensory defensive behavior modulated by neurotransmitter systems associated with fear and stress regulation. However, methodological heterogeneity and developmental variability complicate direct comparisons across studies. Understanding the relationship between IR and fear-related neural circuits could therefore provide valuable insight into how defensive immobility is integrated within broader emotional and cognitive processes. In the present study, we sought to examine the interaction between learned fear and immobility behaviors by inducing an auditory conditioned fear and assessing its effects on two distinct forms of immobility response. Moreover, anxiety-related behaviors were assessed in the Light/Dark Box (LDB) and locomotion in the open field test (OFT). Furthermore, we evaluated potential neuroarchitectural changes in the IL and PrL cortices to explore whether fear-conditioning-induced alterations in prefrontal regions correspond to changes in immobility expression. This approach aims to elucidate the neural mechanisms linking fear regulation and defensive immobility and to provide further insight into adaptive and maladaptive emotional responses..

## Materials and methods 

### Animals and housing

Male (*n* = 16) Wistar rats weighting 230–250 g were obtained from the National School of Biological Sciences (IPN, Mexico). The number of animals used in the present study were based on a priori sample. The rats were housed at constant temperature and humidity (20 °C and 40% relative humidity), with a 12-hour light/dark cycle and food and water *ad libitum*. Three to four rats were housed per standard cage. All procedures complied with the National Institutes of Health Guide for the Care and Use of Laboratory Animals, the technical guidelines for animals in the laboratory issued by SAGARPA Mexico (NOM-062 ZOO-1999), ARRIVE guidelines (Kilkenny et al. 2010) and approved by the National School of Biological Sciences Ethics and Biosecurity Committee (ZOO-008–2025).

### Auditory conditioned fear design

Auditory conditioned fear was induced using a Skinner box, a white chamber (23*12*20 cm) (JP inglobal, model JS1022). Each session included four tone–shock pairings (20-s tone with 0.7 mA, 75–85 dB, frecuency 2–4 kHz, 2-s shock; 120-s intervals) for 30 days (Aguilar et al. 2003). After 24 h of the last exposure, the rats were placed in the training chamber and freezing behavior was monitored for 10 min. In the next sections of the manuscript, we are going to describe this group as conditioned animals. Control animals were exposed only to the tone, without shock.

### Behavioral testing

After the conditioning, animals follow a series of behavioral tests in the following order: Two-compartment LDB, OFT, Clamping the neck and DIR.

### Two-compartment LDB

Anxiety-like behavior was assessed using the LDB test, which relies on the natural aversion of rodents to brightly illuminated areas and their spontaneous exploratory activity. The apparatus consisted of a rectangular box divided into two equal compartments (29 × 30 × 30 cm) connected by a small opening. One compartment was brightly illuminated and painted white, whereas the other was dimly lit and painted black. After 120 s habituation, rats were placed in the center, and time spent in each compartment was recorded for 15 min. Crossings were defined when all four paws entered a compartment (Ennaceur and Chazot 2016).

### OFT

This test was conducted in a square arena (60 × 60 × 30 cm) constructed of black-painted wood with a black floor and no top lid. Each animal was placed individually in the center of the arena, and behavior was recorded for 30 min. A video-tracking system (Videomex V, Columbus Instruments) recorded various behaviors including total distance traveled, ambulation time, stereotypy time, and total rest time. Stereotypy was defined as repetitive, invariant motor patterns such as localized head movements, repetitive sniffing, or grooming sequences occurring within a restricted area. These behaviors were detected automatically by the Videomex-V system based on pixel-level motion patterns confined. The apparatus was cleaned with a 70% ethanol solution between trials to eliminate olfactory cues (Iannitti et al. 2020).

### Clamping the neck

IR was induced in all 16 rats using two methods (Clamping the neck and DIR), applied in the order described below. Each animal underwent one trial per method, with a 1-min intertrial interval. This procedure was induced by applying a clamp (a 5-cm alligator clip with its tips covered in masking tape to prevent skin injury) to the dorsal neck region, specifically between the base of the skull and the posterior margin of the ears. The clamp exerted a pressure of approximately 1300 g/cm² over an area of 1 cm². To enhance the pressure in this region, a second clamp was placed on the ventral aspect of the neck. Following clamp placement, the experimenter manually grasped the animal around the thorax, swiftly inverted it, and gently pressed it onto its back until struggling ceased (approximately 2 s). The experimenter’s hand was then carefully withdrawn, marking the onset of immobility. The rat was placed laterally on a flat surface, and immobility duration was recorded until recovery or 180 s (Zamudio et al. 2009).

### DIR

To induce DIR, each animal was positioned in a V-shaped trough, rapidly inverted, and restrained in the supine position for 15 s. Following this period, the manual restraint was released, marking the onset of the immobility phase. The duration of tonic immobility was recorded from the moment the restraint was removed until escape-like movements occurred or 180 s elapsed; trials without induction were scored as zero (Meyer et al. 1984).

### Golgi-Cox stain

24 h after behavioral testing, rats were anesthetized using a ketamine/xylazine cocktail and subsequently perfused intracardially with 0.9% saline solution. The timing was identical for all animals. Brains were rapidly extracted and processed using a modified Golgi-Cox staining method, as previously described (Flores et al. 2015, 2016; Monroy et al. 2025). The brains were stored in the dark in the Golgi-Cox solution for two weeks and then in a 30% sucrose solution for 3 days. Coronal sectioned, were obtained by using a vibratome (Campden Instrument, MA752, Leicester, UK). Coronal sections (200 μm thick) of the mPFC (Bregma + 3.70 to + 1.70) were obtained using a vibratome in accordance with Paxinos and Watson (1997) coordinates, and mounted on gelatin-coated slides. Four sections per slide were collected on clean, 2% gelatin-coated glass microscope slides. Sections were treated with ammonium hydroxide for 30 min, immersed in Kodak film fixer for 30 min, rinsed with distilled water, dehydrated through graded alcohols, and mounted with a resinous medium.

Neuronal morphology was analyzed using a light microscope (DM2000, Leica Microsystems, USA) equipped with a camera lucida. Ten pyramidal neurons per brain (five per hemisphere) were reconstructed in two dimensions from the PrL and IL regions (layer III) of the mPFC. Dendritic architecture was assessed using Sholl analysis (Sholl 1953), employing a transparent grid with concentric circles spaced 10 μm apart to estimate the number of dendritic intersections. Total dendritic length (TDL) was calculated by multiplying the number of intersections at each radius by 10 μm. Additionally, the number of dendritic branches was quantified across successive branch orders from the soma. Spine density was evaluated by drawing ten distal or terminal dendritic segments (approximately 10 μm in length; five per hemisphere) at 1000× magnification and quantifying the number of spines per segment.

### Statistical analysis

Behavioral and morphological data were expressed as mean ± SEM and analyzed using GraphPad Prism 8. Comparisons of locomotion activity, immobility, TDL, and spine density were performed using Student’s t-test. LDB data were analyzed by one-way ANOVA followed by Sidak’s post hoc test. Sholl and dendritic order analyses were conducted using two-way ANOVA. Statistical significance was set at *P* < 0.05.

## Results

### Conditioned rats displayed higher freezing

**Fig. 1:**
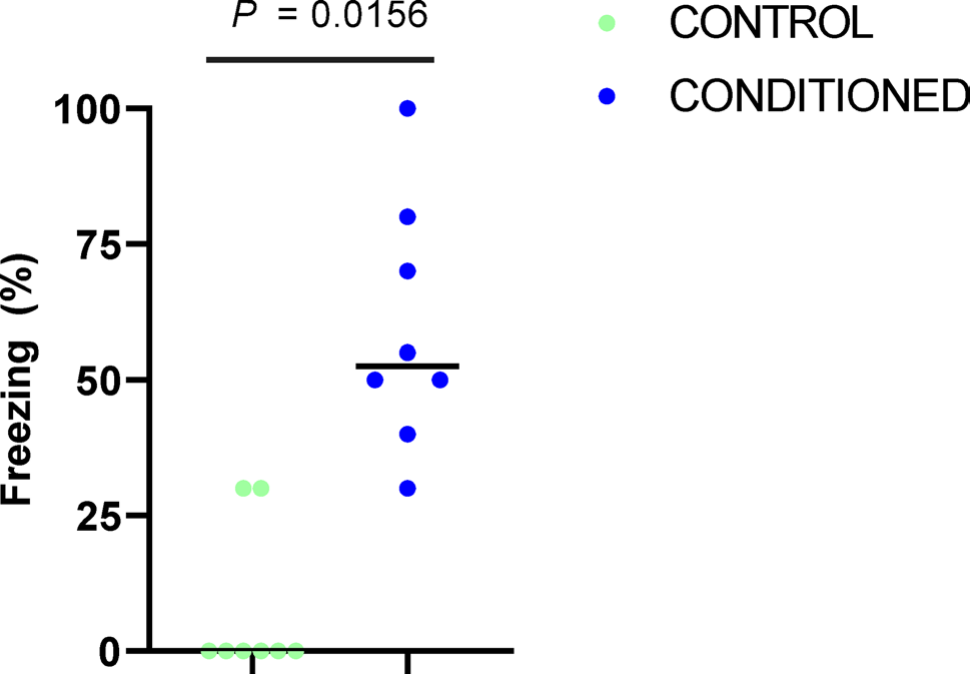
Comparison of freezing between control and conditioned rats after conditioning protocol. Conditioned rats spent more time freezing compared to control animals. Data are presented as mean ± SEM, *n* = 8 per group, *P < 0.05

The freezing time was significantly increased in conditioned rats (*t* = 5.50, *df* = 14, *P* = 0.0156) (Fig. 1).

### Conditioned rats spent less time in the bright compartment in the LDB

**Fig. 2:**
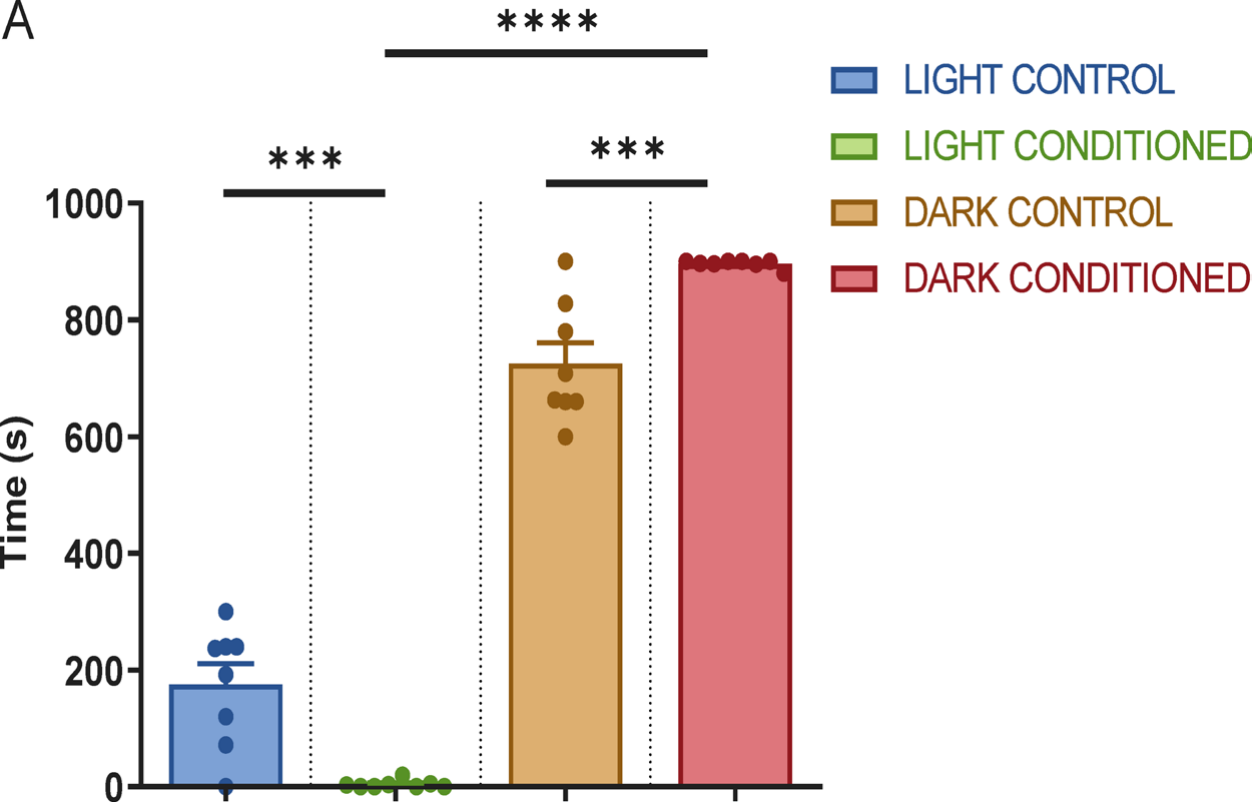
Time spent in the compartments of the light/dark box by control and conditioned rats. **A.** Comparison of the time spent (in seconds) in either the light or dark compartment between control and conditioned rats. Data are presented as mean ± SEM, *n* = 8 per group, ****P < 0.0001, ***P < 0.001

Conditioned rats spent significantly less time in the light compartment compared to controls (one-way ANOVA, F(3,28) = 281.6; post hoc, *P* < 0.0002) and correspondingly more time in the dark compartment (one-way ANOVA, F(3,28) = 281.6; control vs. conditioned, light/dark, *P* = 0.0002) (Fig. 2). Overall, all rats exhibited a strong preference for the dark compartment (one-way ANOVA, F(3,28) = 281.6; *P* < 0.0001). In addition, conditioned rats displayed reduced exploratory behavior, remaining mostly confined to a single corner within the dark compartment.

### Conditioned rats displayed reduced locomotor activity in the OFT

**Fig. 3:**
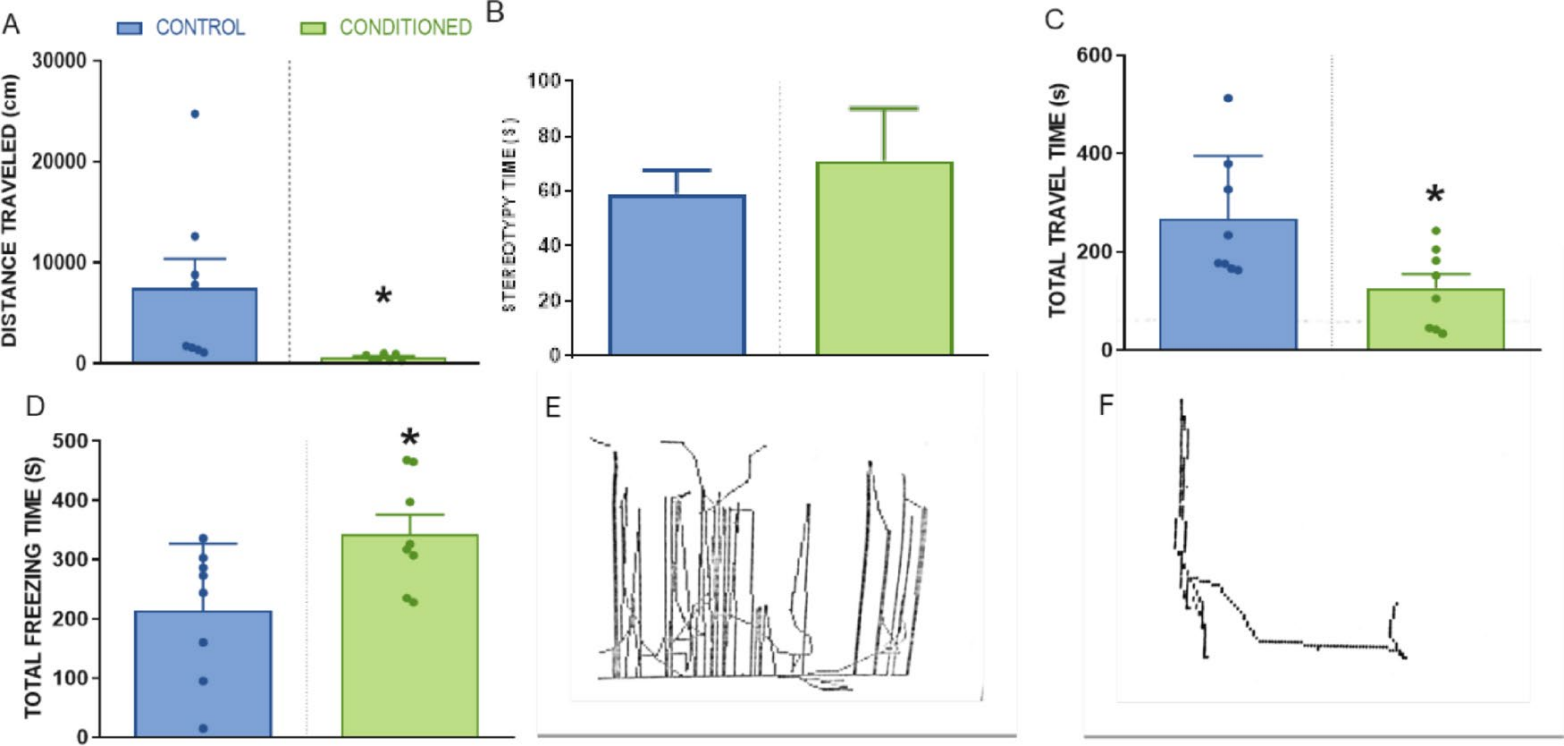
Comparison of locomotor activity in the open field test between control and conditioned rats. **A.** Distance traveled (cm), **B.** Time spent in stereotyped behaviors (s), **C.** Ambulation time (s), **D.** Resting time (s). Representative locomotor paths in this test with example of a path traced by a control rat (**E**) and a conditioned rat (**F**). Data are presented as mean ± SEM, *n* = 8 per group, *P < 0.05

Locomotor activity in the OFT revealed a reduction in spontaneous exploratory behavior in rats exposed to novel and open contexts. This effect was supported by statistically significant differences in three of the four analyzed variables: total distance traveled (*t* = 2.358, *df* = 14, *P* = 0.0334) and ambulation time (*t* = 2.624, *df* = 14, *P* = 0.0200), both of which showed a significant decrease in conditioned rats. Conversely, resting time was significantly increased (*t* = 2.493, *df* = 14, *P* = 0.0258). No significant differences were observed in stereotypy time between control and conditioned groups (*t* = 0.5752, *df* = 14, *P* = 0.5743) (Fig. 3).

### Conditioned rats displayed increased immobility in the DIR but not in the clamping the neck

**Fig. 4:**
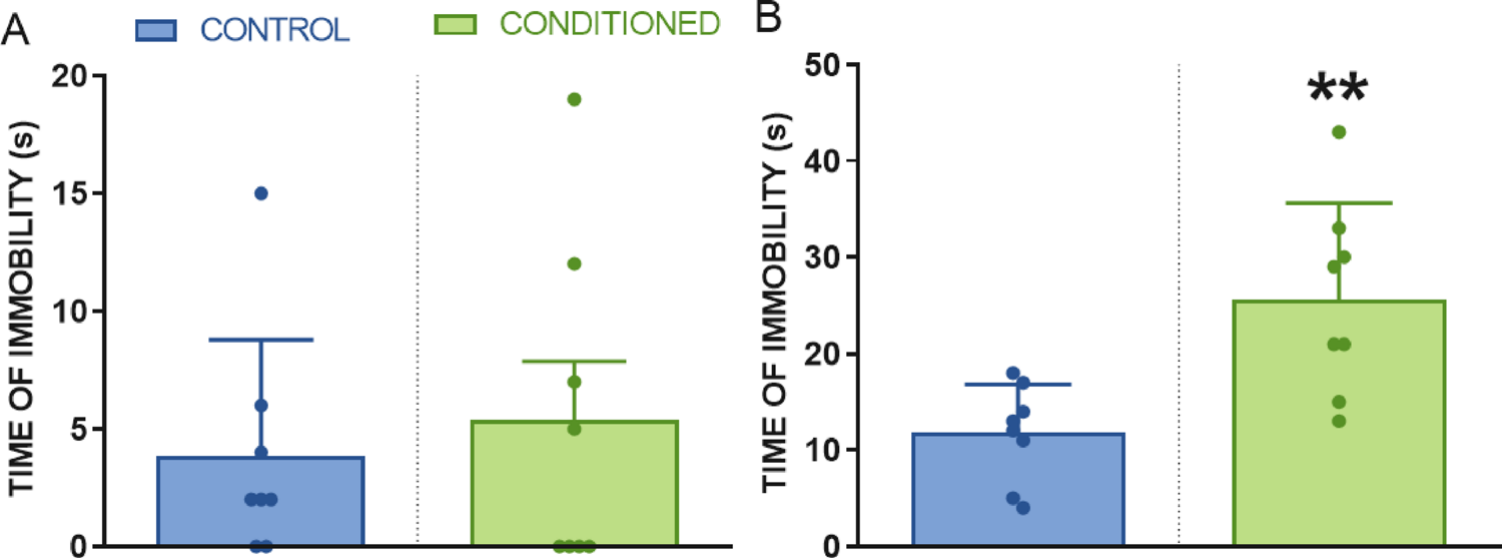
Comparison of immobility duration in the neck-clamp test and dorsal immobility response between control and conditioned rats. In the neck-clamp test, there were no significant differences between groups (**A**). In the dorsal immobility response, there were an increase in the time the rat was immobile in the conditioned group (**B**). Data are presented as mean ± SEM. *n* = 8 per group, **P < 0.01

Immobility was assessed using both the DIR and the dorsal neck-clamping test. No significant differences were detected in the DIR test between conditioned and control rats (t = 0.494, df = 14, *P* = 0.6291) (Fig. 4A). In contrast, the DIR revealed a significant increase in immobility duration in conditioned (phobic) rats compared to non-conditioned controls (t = 3.497, df = 14, *P* = 0.0036) (Fig. 4B).

### Conditioned rats increased arborization and spine density in the PrL cortex

**Fig. 5:**
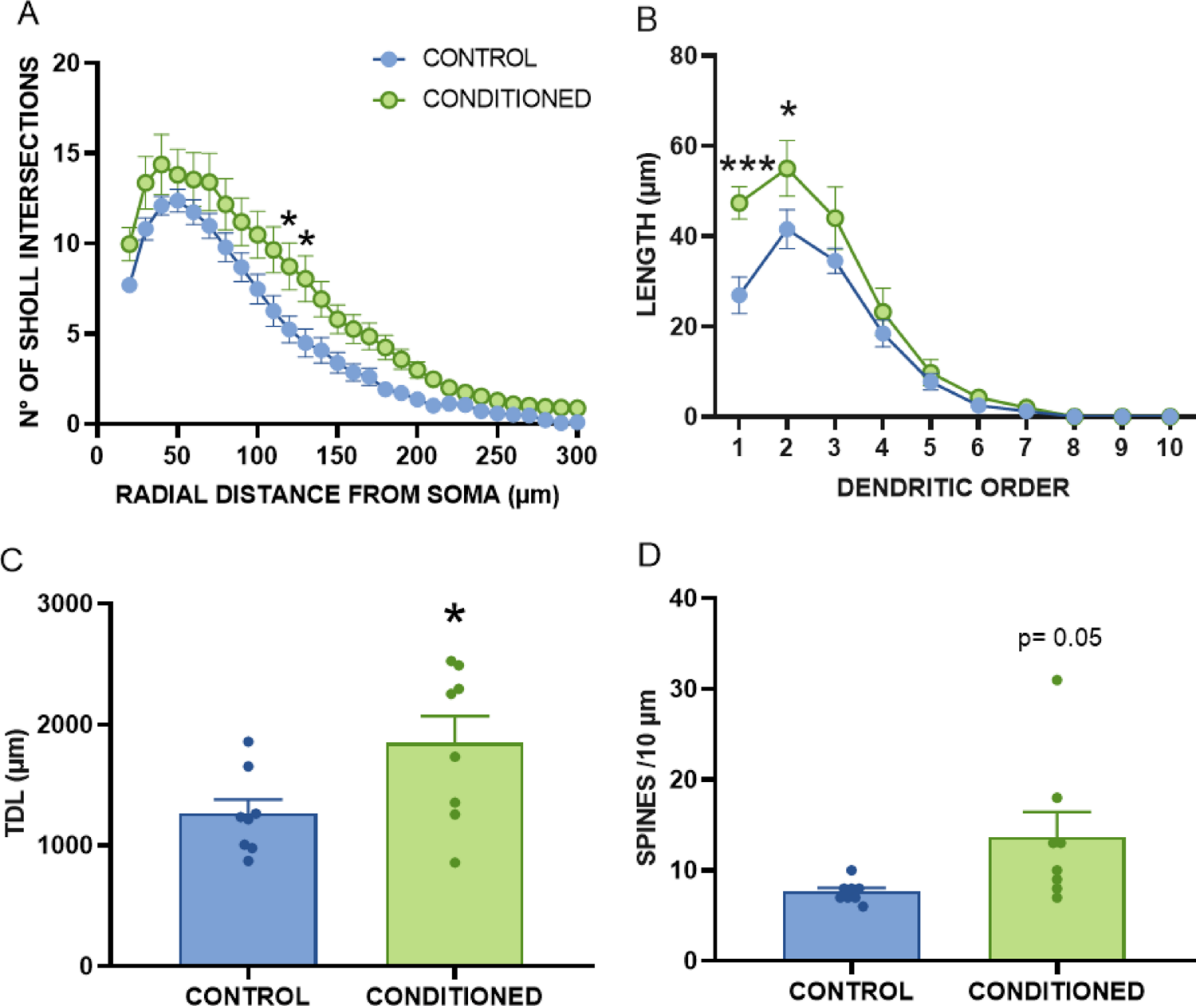
Conditioned rats increased arborization in the prelimbic cortex. Intersections of dendrites indicate increase close to the soma at 120 and 130 μm in neurons of this brain region in conditioned rats relative to controls (**A**). Branch length was augmented in first and second order (**B**). Total dendritic length was increased in conditioned versus control animals (**C**). The number of spines per segment was at the threshold for significance (**D**). Data are mean ± SEM with *n* = 8 rats per group, 8–10 neurons per dendritic segment were drawn for each animal. *P < 0.05, ***P < 0.001

Sholl analysis revealed a significant increase in dendritic arborization (number of intersections per dendritic length in µm) in conditioned rats at 120 and 130 μm from the soma) (two-way ANOVA, treatment *F*(1,14) = 4.524, *P* ≤ 0.05, distance to the soma F(1.744, 24.41) = 183.1, *P* ≤ 0.001; Interaction F(28, 392) = 1.975, *P* ≤ 0.001) (Fig. 5A). Within the conditioned group, dendritic arborization showed significant differences in the number of intersections as a function of distance from the soma, with an increase in this region (two-way ANOVA, treatment *F*(1,14) = 4.373, *P* ≤ 0.05, distance to the soma F(2.272, 31.81) = 97, *P* ≤ 0.001; Interaction F(9, 126) = 3.32, *P* ≤ 0.001) (Fig. 5B). Condition led to a significant increase in TDL in the PrL region compared to controls (t-test, P ≤ = 0.03) (Fig. 5C). Statistical analysis revealed an increase in spine density in PrL neurons of conditioned rats compared to controls (t-test, *P* = 0.05) as shown in Fig. 5D.

### Conditioned rats decreased arborization and spine density in the IL cortex

**Fig. 6:**
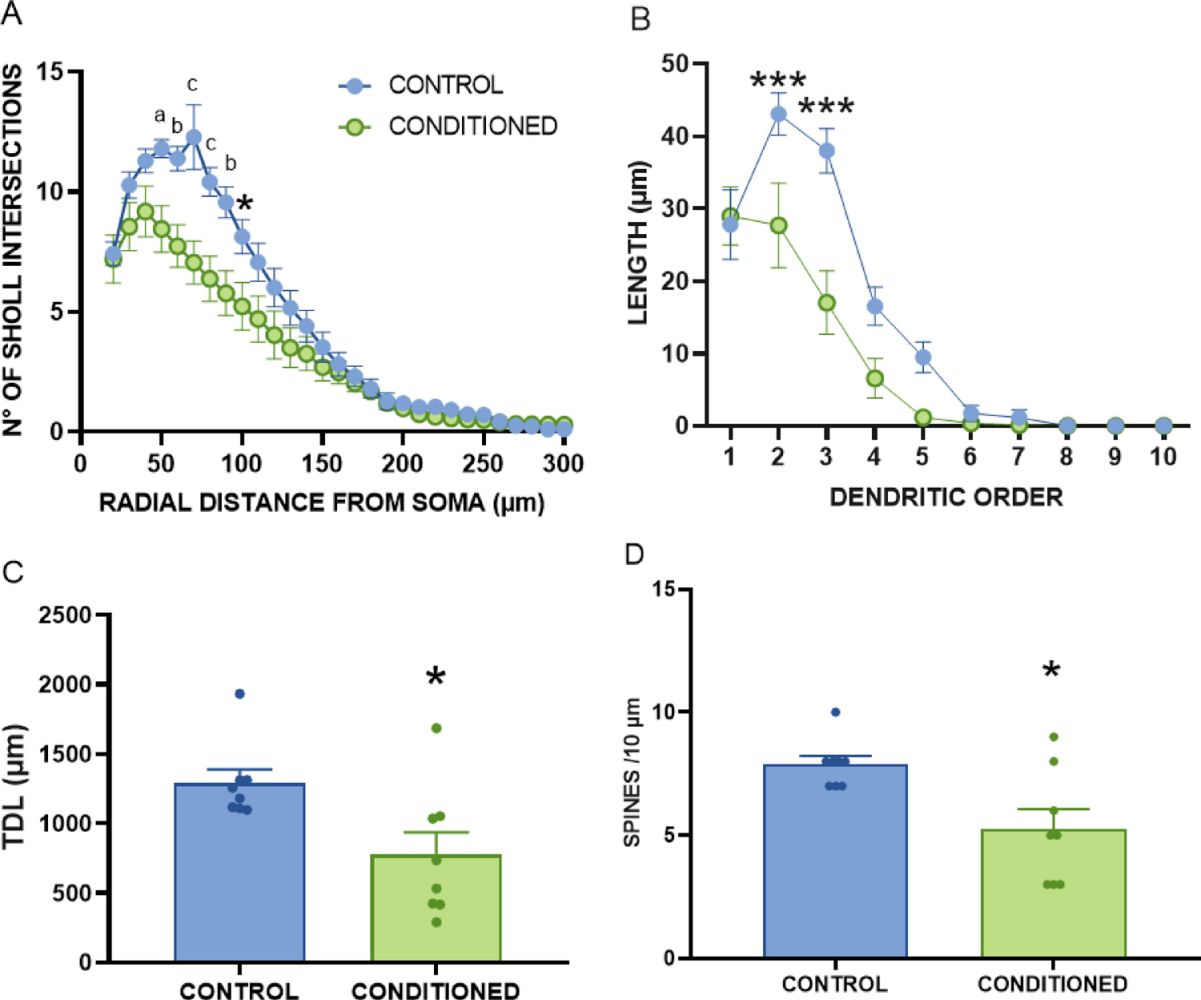
Conditioned rats decreased arborization and spine density in the infralimbic cortex. Intersections of dendrites are decreased close to the soma in neurons of this brain region in conditioned rats relative to controls (**A**). Branch length was reduced in second and third order (**B**). Total dendritic length was decreased in conditioned versus control animals (**C**). The number of spines per segment was reduced in neurons of conditioned animals (D). Data are mean ± SEM with *n* = 8 rats per group, 8–10 neurons per dendritic segment were drawn for each animal. *P < 0.05; ***P < 0.001; a *P* < 0.01; b *P* < 0,001; c *P* < 0,0001

Conditioned rats presented a significant reduction in dendritic arborization as indicated by the Sholl analysis, measured as the number of intersections per dendritic length (µm), at multiple distances from the soma (two-way ANOVA: treatment F(1,14) = 5.14, *P* ≤ 0.05; distance to soma F(2.886, 40.41), *P* ≤ 0.001; interaction F(28,392) = 5.872, *P* ≤ 0.001) (Fig. 6A). In the conditioned group, dendritic arborization varied significantly with distance from the soma, showing a decrease in the proximal region (two-way ANOVA: treatment F(1,14) = 9.009, *P* ≤ 0.001; distance F(2.087,29.21) = 72, *P* ≤ 0.001; interaction F(9,126) = 5.529, *P* ≤ 0.001) (Fig. 6B). Conditioned rats also exhibited a significant decrease in TDL compared with controls (t-test, *P* = 0.0169) (Fig. 6C). Furthermore, spine density was significantly decreased in conditioned rats than in controls (t-test, *P* = 0.0106) (Fig. 6D).

## Discussion

Conditioned rats exhibited pronounced behavioral alterations, increased freezing without the shock, spending less time in the light compartment and more time in the dark compartment of the LDB, suggesting anxiety-related behavior. In the OFT, these rats showed reduced locomotor activity alongside increased freezing behavior. Similarly, in the DIR, conditioned rats displayed elevated immobility, whereas no differences were observed in the neck-clamp test. Morphological analyses revealed region-specific changes in the PFC: conditioned rats showed hypertrophy in the PrL region, while the IL cortex exhibited hypotrophy and reduced spine density. These opposing, region-dependent effects are both novel and noteworthy, suggesting differential cortical adaptations associated with the conditioning protocol.

### Conditioned rats displayed anxiety-related behavior in the LDB

The LDB test is based on the innate aversion of rodents to illuminated areas, which consistently drives their preference toward the dark compartment (Ennaceur and Chazot 2016). In the present study, conditioned rats spend less time in the light area, suggesting anxiety-related behavior. Chaouloff et al. (1997) reported that rats exhibiting high levels of anxiety displayed reduced exploratory behavior in novel environments, along with a pronounced preference for the dark compartment—a behavioral pattern also observed in the conditioned rats in this study. Similarly, Campos et al. (2013) emphasized that this paradigm reflects the intrinsic conflict in anxious rats between exploratory drive and avoidance of the illuminated compartment. These results indicate that the conditioned rats developed an anxiety-like phenotype characterized by heightened avoidance of illuminated areas.

### Conditioned rats presented anxiety-related behavior in the OFT

Locomotor activity in the OFT is defined as the movement of an organism from one location to another and represents a fundamental aspect of animal behavior, playing a critical role in survival and adaptive responses (Kraeuter et al. 2019). In this study, we observed that conditioned rats displayed reduced locomotion in this paradigm. Animals subjected to chronic stressors often exhibit reduced movement in open environments, where they perceive potential external threats (Wang et al. 2021). This behavioral alteration may be influenced not only by diminished motivation but also by physical manifestations of anxiety, such as fatigue and muscle tension, which contribute to the observed decrease in locomotor activity. Early on, Katz et al. (1981) reported that rats subjected to chronic stress exhibited reduced locomotor activity in the open field, particularly within the central zone of the arena. Notably, administration of pargyline, a monoamine oxidase B (MAO-B) inhibitor, mitigated these effects by enhancing exploratory behavior, increasing entries into the central zone, and reducing defecation. The authors attributed these behavioral improvements to the concomitant reduction in plasma corticosterone levels observed in treated animals relative to stressed, vehicle-treated controls. The conditioned rats in the present study showed behavioral patterns similar to stressed animals indicating anxiety-related behavior, likely as a strategy to avoid potential external threats.

### Immobility responses as indicators of fear and anxiety

While conditioned rats displayed elevated immobility in the DIR, no differences were observed in the neck-clamp test. Both tests are based on instinctive defensive behaviors triggered by perceived threats, during which the animal remains motionless and unresponsive to external stimuli, mimicking death. These paradigms are therefore widely used to evaluate fear- and anxiety-related responses. de la Cruz and Junquera (1993) who compared three immobility paradigms—DIR, bandaging, and clamping the neck—reported that immobility duration was significantly longer in the DIR. Moreover, this immobility remained stable even when animals were subjected to a second trial cycle, in contrast to pinching immobility, which showed an abrupt reduction within the first 40 days of experimentation before increasing again in later trials. If immobility is considered an antipredatory strategy, one explanation for the greater effectiveness of dorsal grasping may lie in the continuous and close physical presence of the experimenter, which is inherent to the procedure. Another relevant factor is that, unlike clamping the neck or bandaging, DIR ends specifically when the animal initiates escape-related movements, with no ambiguity in the termination point of the response (de la Cruz and Junquera 1993). In agreement, Meyer and Bohus (1983) described DIR as resembling “the posture of a prey when carried by a predator,” which may account for the prolonged immobility observed in conditioned rats. Similarly, de-la-Cruz et al. (1987) emphasized immobility as an adaptive behavior, since predators typically react to live, moving prey but often lose interest in immobile prey that appear dead. Further supporting this view, Zamudio et al. (2009) linked immobility responses to hypothalamic-pituitary-adrenal (HPA) axis activity. They reported that prior stress exposure—such as tail heating or placement in a novel elevated open environment—increased immobility duration in the pinching test. In addition, corticosterone injections mimicking plasma levels observed during stress also prolonged immobility responses. This study reinforced the interpretation of immobility as a defensive behavior, in which both prior and concurrent stressors can amplify the duration of the response.

### Conditioned rats showed hypertrophy in PrL neurons and hypotrophy in IL neurons

In the present study, we observed that conditioned rats showed neuronal hypertrophy in the PrL region and neuronal hypotrophy in the IL cortex. The PrL and IL cortices are subregions of the mPFC in rodents, and they play distinct but complementary roles in the regulation of fear and anxiety (Quirk and Vidal-Gonzalez 2006; Sierra-Mercado et al. 2011; Likhtik and Paz 2015). Their functions are often compared to the dorsal anterior cingulate cortex (ACC) and ventromedial prefrontal cortex (vmPFC) in humans, respectively.

Our results indicate that conditioned rats display neuronal hypertrophy in the PrL cortex. In particular, the PrL cortex is activated during retrieval and expression of conditioned fear and sends excitatory outputs to the BLA (Likhtik and Paz 2015). Moreover, hyperactivity or hyperconnectivity of the PrL is associated with anxiety-like behaviors and persistent fear memories (Sotres-Bayon et al. 2012; Gao et al. 2023; Smoak et al. 2025). Sierra-Mercado et al. (2011) observed that activity of this cortical area is necessary for fear expression but not extinction memory. Inactivation of both the PrL and IL cortices impaired fear expression and extinction (Sierra-Mercado et al. 2006). In contrast, mice undergoing uncontrollable stress and deficits in fear extinction do not present modifications in dendritic morphology in PrL cortex (Izquierdo et al. 2006).

Our results indicate that conditioned rats display neuronal hipothophy in the IL cortex. This cortex is connected to the hippocampus and its activation is essential for extinction learning and recall. In agreement with current findings, mice undergoing uncontrollable stress and deficits in fear extinction display dendritic reduction in IL neurons (Izquierdo et al. 2006). Moreover, reduced IL activity correlates with impaired fear extinction and pathological anxiety (Sierra-Mercado et al. 2011). This region suppresses fear expression by inhibiting activity in the central amygdala (CeA), whereas the PrL facilitates fear responses through excitatory projections to the basolateral amygdala (BLA) and CeA (Vidal-Gonzalez et al. 2006). Therefore, the dynamic interaction between the PrL and IL cortices may determine whether fear is expressed or suppressed. When PrL activity predominates, fear expression and anxiety are observed, whereas dominance of the IL cortex is associated with emotional flexibility.The current neuromorphological results may explain why the conditioned rats developed an anxiety-like phenotype in the LDB test and OFT. Regarding the immobility results, Lalonde and Strazielle (2022) showed different subcortical brain structures involved in the control of immobility reflexes. While the tests are similar, the neuronal substrate are not identical, therefore, we observe different results.

## Conclusions

Conditioned rats developed a clear anxiety-like phenotype, characterized by avoidance of illuminated areas, reduced exploratory locomotion, and increased immobility in defensive paradigms. These behavioral alterations were accompanied by distinct, region-specific morphological adaptations in the PrL and IL cortices. This opposing pattern suggests an imbalance in the PrL–IL circuitry, favoring fear-related expression and anxiety-like behavior over emotional regulation and flexibility. Together, the present findings indicate that the conditioning protocol induces both behavioral and cortical adaptations that mirror key aspects of pathological anxiety, providing a relevant model for studying the neural basis of fear-related disorders.

## Data Availability

Data should be shared upon reasonable request.
